# Interventional treatment for acute cerebral infarction with large vessel occlusion combined with aortic arch interruption: A case report

**DOI:** 10.1097/MD.0000000000027879

**Published:** 2021-11-19

**Authors:** WenSheng Zhang, WeiFang Xing, MinZhen Zhu, XiaoJing Zhong, JinZhao He

**Affiliations:** Department of Neurology, Heyuan People's Hospital, Guangdong Provincial People's Hospital Heyuan Hospital, Guangdong Province, China.

**Keywords:** acute cerebral infarction, aortic arch interruption, case report, interventional treatment, large vessel occlusion

## Abstract

**Rationale:**

Aortic arch interruption is a type of congenital vascular malformation that is often observed in childhood. Most children die of congestive heart failure due to rapid deterioration. Children can only survive to adulthood if they have extremely rich collateral circulation. Cases of acute cerebral infarction with large vessel occlusion receiving interventional treatment in adult patients with interrupted aortic arch have not been reported.

**Patient concerns:**

A 55-year-old man with a history of atrial fibrillation and smoking but without a family history of stroke was admitted to our hospital with a 5-hour history of left limb weakness and speech difficulties.

**Diagnoses:**

Emergency brain computed tomography showed a large cerebral infarction in the right frontal temporal parietal lobe. He was suspected to have aortic arch interruption in the early stage of endovascular interventional therapy through the femoral artery approach, and was converted to the transradial artery pathway. The aortic arch was disconnected, and the right internal carotid artery was occluded.

**Interventions:**

Considering the possibility of cardiogenic embolism, a middle catheter was used for thrombus aspiration of the right internal carotid artery. After removal of the dark red thrombus was removed, the right internal carotid artery was successfully recanalized.

**Outcomes:**

The patient recovered well after the operation. However, the patient and his family refused further treatment for aortic arch interruption. The modified Rankin Scale score was 0 at 3 months and 1 year of follow-up which meant that he recovered quite well.

**Lessons:**

Adult patients with acute cerebral infarction with large vessel occlusion are rarely complicated with aortic arch interruption, and emergency thrombectomy via the radial artery approach is feasible.

## 1 Introduction

Aortic arch interruption is a congenital vascular malformation that is often observed in childhood. Most children die from congestive heart failure due to rapid deterioration.^[1]^ Children receiving surgical treatment have a chance to survive for a long time. Without surgical treatment, children only survive to adulthood in the presence of extremely rich collateral circulation.^[2]^ Of course, there are also individual patients who survive to adulthood without any treatment, but such patients are rare. The clinical manifestations of adult patients are different from those of young children, who can be completely asymptomatic and have intractable hypertension, headache, and complications of cerebral aneurysm rupture and hemorrhage.^[3,4]^ However, aortic arch interruption has not been reported in adult patients with cerebral infarction or cerebrovascular occlusion. In such patients, aortic arch interruption is usually found during imaging examinations for other diseases. Here, we present an adult patient who received interventional therapy for acute cerebral infarction with large vessel occlusion complicated with aortic arch interruption.

## 2 Case presentation

A 55-year-old man with a history of atrial fibrillation and smoking but without a family history of stroke was admitted to our hospital with a 5-hour history of left limb weakness and speech difficulties. The heart rhythm was uneven, and the first heart tone was neither strong nor weak. He had mixed aphasia, eye gaze to the right, shallow left nasal and labial groove, mouth angle slanted to the right, left limb muscle strength grade 1, positive left side pathological signs, and National Institutes of Health Stroke Scale (NIHSS) score of 16 points. Emergency brain computed tomography (CT) showed a large cerebral infarction in the right frontal temporal parietal lobe. Chest CT revealed pulmonary edema and double lung inflammation.

**Figure 1:**
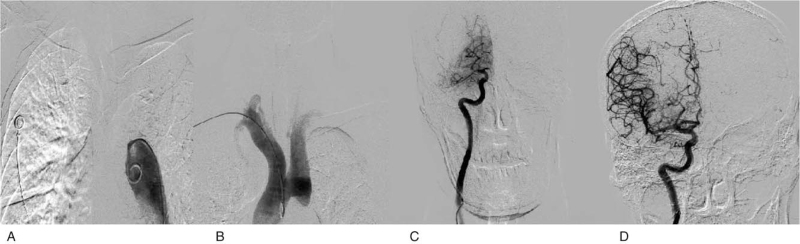
(A) The angiographic guide wire and pigtail catheter could not pass through the aortic arch during cerebral angiography. After changing to the radial approach, aortic arch angiography revealed aortic arch interruption (B) and right internal carotid artery occlusion (C). (D) The right internal carotid artery was successfully recanalized after thrombectomy.

There was a need to recanalize because of cerebrovascular occlusion, and the family members of the patient agreed to undergo endovascular interventional therapy. After successful right femoral artery puncture, the angiography guidewire could not pass through the aortic arch (Fig. 1A). 5F pigtail angiography showed that there was no development of the aortic arch or vessels above the arch. Considering the vascular variation, we performed a right radial artery puncture. Cerebral angiography was performed again and aortic arch interruption was clearly diagnosed (Fig. 1B). The right internal carotid artery was occluded (Fig. 1C), and the posterior communicating artery was opened. Considering the possibility of cardiogenic embolism, a middle catheter was used for thrombus aspiration of the right internal carotid artery, and a dark red thrombus was removed. Angiography showed that the right middle cerebral artery, right anterior cerebral artery trunk and distal branches were clearly visible (Fig. 1D), no luminal stenosis was found, modified thrombolysis in cerebral infarction score forward blood flow was grade 3, and the operation was successful.

**Figure 2:**
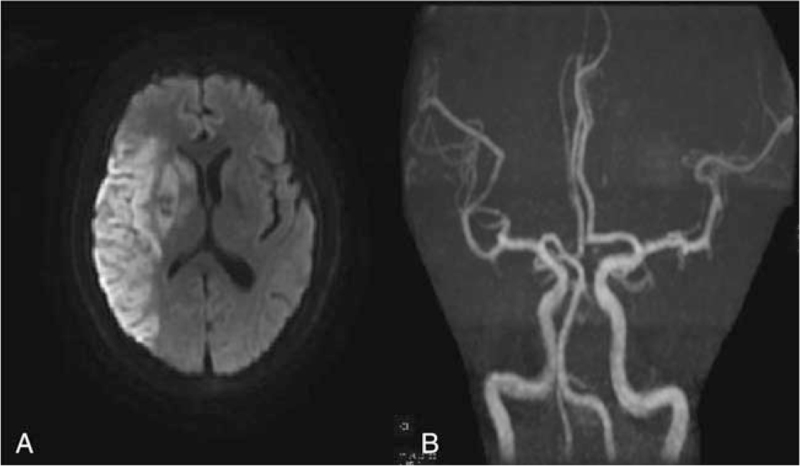
(A) Brain magnetic resonance imaging showed that the right frontal lobe, temporal lobe, occipital lobe, parietal lobe, insular lobe, radial crown and basal ganglia had massive acute cerebral infarction, and the right basal ganglia had small hemorrhage after infarction. (B) The right internal carotid artery was unobstructed.

**Figure 3:**
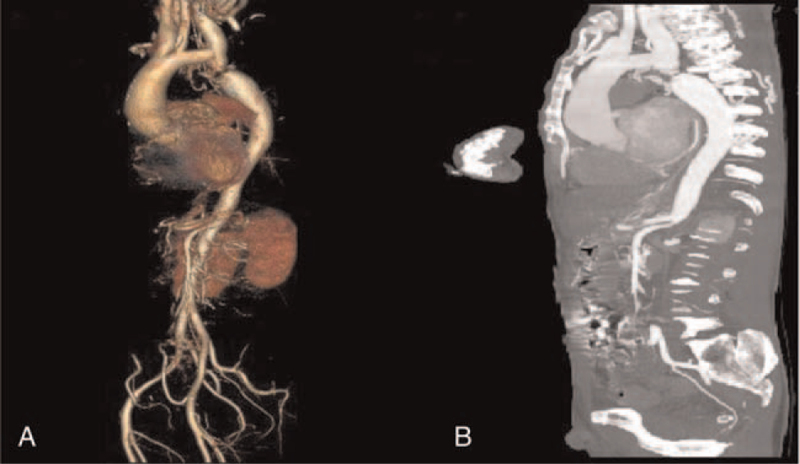
(A, B) Computed tomography angiography of aortic arch confirmed the diagnosis of interrupted aortic arch again.

No hemorrhage was found on brain CT immediately after the operation and on postoperative day 1. Treatment to improve circulation, anticoagulation, control ventricular rate and anti-infection were administered. Unfortunately, oxygen saturation decreased on postoperative day 3, and he underwent tracheal intubation. The NIHSS score decreased to 4 on postoperative day 6. The patient was breathing smoothly on postoperative day 7, and tracheal intubation was successfully performed. Magnetic resonance imaging showed a large area of right-side acute cerebral infarction and small hemorrhage after infarction in the right basal ganglia, and the right internal carotid artery was unobstructed (Fig. 2A, B). Computed tomography angiography indicated that the aortic arch was interrupted (A type, congenital cardiovascular malformation), and multiple collateral vessels were found (Fig. 3A, B). The NIHSS score decreased to 1 point at 1 month after the operation, which satisfied the patient and his family members, and the patient was discharged after improvement of his condition. The patient and his family refused further treatment for aortic arch interruption. The modified Rankin Scale score was 0 at 3 months and 1 year postoperatively, indicating a good prognosis.

## 3 Discussion

Aortic arch interruption is a rare congenital developmental anomaly, which is defined as the complete loss of continuity between the ascending and descending parts of the aorta and is usually associated with heart defects.^[5]^ Most patients die in childhood, but those who receive surgical treatment have a chance of longer survival, but there are few reports about it. Adults are usually found in the process of further examination after the emergence of refractory hypertension.^[6]^ Adult aortic arch amputation has rarely been reported, and its survival can be regarded as unusual. It was first described by Stedel in 1778, and the first classification system was introduced by Celia and Patton in 1959. In this classification, aortic arch interruption can be divided into 3 types according to the location of the aortic discontinuity: type A is located at the distal origin of the left subclavian artery; type B is located between the left common carotid artery and the left subclavian artery; and Type C is located between the innominate artery and left common carotid artery.^[7]^ In neonates, Type B is the most common (53%), which is associated with DiGeorge syndrome and chromosome 22q11.2 deletion, whereas Type A is more common in adults (79%).^[7]^ In patients with aortic arch interruption, survival to adulthood is rare and even rarer to live to 50 years without surgical intervention, which depends on abnormally rich collateral circulation.

Most cases of aortic arch interruption are diagnosed in the neonatal period, accompanied by severe congestive heart failure, and the clinical symptoms of children worsen rapidly. Without timely treatment, 90% of the affected infants die in the first year of life, and most of them die in the first few days.^[8]^ In a few case reports, the clinical manifestations of adult patients with aortic arch interruption are significantly different from those of children. Adults may not have any symptoms, but also have refractory hypertension, headache, claudication, large differences in limb blood pressure, congestive heart failure, and recurrent cerebral infarction.^[9,10]^ At present, there are no report of adult patients with aortic arch interruption combined with acute cerebral infarction and cerebral large vascular occlusion. This case report presents a case of aortic arch interruption combined with right internal carotid artery occlusion. The patient survived after an emergency endovascular intervention, with an acceptable prognosis. Acute cerebral infarction with large vessel occlusion is a rare clinical manifestation in patients with interrupted aortic arch, but there is no evidence to prove that there is a direct relationship between interrupted aortic arch and acute large vessel occlusion and cerebral infarction.

In the present case, an aortic arch interruption was found by accident during cerebral angiography. The operator identified the aortic arch interruption and switched to the radial artery approach for endovascular interventional therapy. The occluded right internal carotid artery was opened, which was a key factor for the patient to benefit from endovascular interventional therapy. This case also reminds all the neurointerventional physicians that, in emergency interventional recanalization of large cerebral vascular occlusion, a small number of patients have aortic arch interruption. If it is found that the catheter is difficult to pass through the aortic arch, the possibility of aortic arch interruption should be considered, and the path should be changed in time to reduce the delay of endovascular interventional recanalization of acute cerebral infarction with large cerebral vascular occlusion in order to increase the possibility of these patients benefiting from endovascular interventional therapy.

For patients with aortic arch interruption who survive to age 50 years, there is insufficient evidence for aortic arch reconstruction at present, because the treatment of aortic arch interruption recanalization with stent implantation or other methods is difficult and risky, but there is no clear recommendation for conservative drug treatment.^[11]^ After the recovery of symptoms of acute cerebral infarction, our patient and his family refused further treatment for aortic arch interruption and took antiplatelet drugs after discharge. The modified Rankin Scale score was 0 at 3 month and 1 year follow-up, indicating that adult patients with aortic arch interruption can benefit from endovascular interventional therapy when they also have acute cerebral infarction with large vessel occlusion.

## 4 Conclusion

Our case report suggests that there are still some patients with interrupted aortic arch who can survive for a long time even without aortic arch reconstructive surgery. However, once adult patients with aortic arch interruption have acute cerebral infarction due to large vessel occlusion and cannot be operated upon through the femoral artery approach, it is feasible to change to the transradial artery approach for emergency endovascular intervention.

## Acknowledgments

The authors thank Yangchun Wen and Guanghong Zhong for their assistance in the preparing of the manuscript.

## Author contributions

**Data curation:** WenSheng Zhang, WeiFang Xing.

**Investigation:** WenSheng Zhang, MinZhen Zhu.

**Methodology:** WenSheng Zhang, XiaoJing Zhong.

**Project administration:** WenSheng Zhang.

**Supervision:** JinZhao He.

**Writing – original draft:** WenSheng Zhang, WeiFang Xing.

**Writing – review & editing:** WenSheng Zhang, WeiFang Xing.
